# Reliability of two behavioral tools to assess pain in preterm neonates

**DOI:** 10.1590/S1516-31802003000200008

**Published:** 2003-03-05

**Authors:** Ruth Guinsburg, Maria Fernandes Branco de Almeida, Clóvis de Araújo Peres, Alexandre R. Shinzato, Benjamin Israel Kopelman

**Keywords:** Pain, Newborn, Infant, Assessment, Dor, Recém, Nascido, Avaliação

## Abstract

**CONTEXT:**

One of the main difficulties in adequately treating the pain of neonatal patients is the scarcity of validated pain evaluation methods for this population.

**OBJECTIVE:**

To analyze the reliability of two behavioral pain scales in neonates.

**TYPE OF STUDY:**

Cross-sectional.

**SETTING:**

University hospital neonatal intensive care unit.

**PARTICIPANTS:**

22 preterm neonates were studied, with gestational age of 34 ± 2 weeks, birth weight of 1804 ± 584 g, 68% female, 30 ± 12 hours of life, and 30% intubated.

**PROCEDURES:**

Two neonatologists (A and B) observed the patients at the bedside and on video films for 10 minutes. The Neonatal Facial Coding System and the Clinical Scoring System were scored at 1, 5, and 10 minutes. The final score was the median of the three values for each observer and scale. A and B were blinded to each other. Video assessments were made three months after bedside evaluations.

**MAIN MEASUREMENTS:**

End scores were compared between the observers using the intraclass correlation coefficient and bias analysis (paired t test and signal test).

**RESULTS:**

For the Neonatal Facial Coding System, at the bedside and on video, A and B showed a significant correlation of scores (intraclass correlation score: 0.62), without bias between them (t test and signal test: p > 0.05). For the Clinical Scoring System bedside assessment, A and B showed correlation of scores (intraclass correlation score: 0.55), but bias was also detected between them: A scored on average two points higher than B (paired t test and signal test: p < 0.05). For the Clinical Scoring System video assessment, A and B did not show correlation of scores (intraclass correlation score: 0.25), and bias was also detected between them (paired t-test and signal test: p < 0.05).

**CONCLUSION:**

The results strengthen the reliability of the Neonatal Facial Coding System for bedside pain assessment in preterm neonates.

## RESUMO

**CONTEXTO:**

Uma das principais dificuldades para tratar adequadamente a dor no recémnascido é a pobreza de métodos validados para avaliar a dor nessa faixa etária.

**OBJETIVO:**

Analisar a confiabilidade de duas escalas comportamentais de avaliação da dor do recém-nascido.

**TIPO DE ESTUDO:**

Estudo transversal.

**LOCAL:**

Unidade de terapia intensiva neonatal de hospital universitário.

**PARTICIPANTES:**

22 prematuros com idade gestacional de 34 ± 2 semanas, peso ao nascer de 1.804 ± 584 gramas, 68% femininos, com 30 ± 12 horas de vida e 30% com intubação traqueal no momento do estudo.

**PROCEDIMENTOS:**

Dois neonatologistas (A e B) observaram os recém-nascidos à beira do leito e em filmes de vídeo durante 10 minutos. O Sistema de Codificação da Atividade Facial Neonatal e a Escala de Conforto Clínico foram aplicados 1, 5 e 10 minutos após o início da observação. A pontuação final foi obtida pela mediana dos três valores, para cada observador e para cada escala. O neonatologista A desconhecia a avaliação de B e vice-versa. A análise dos filmes de vídeo foi realizada da mesma maneira, três meses após a avaliação à beira do leito.

**VARIÁVEIS ESTUDADAS:**

Os escores finais foram comparados entre os observadores por meio do cálculo do coeficiente de correlação intraclasse e por análise da presença de viés (teste t pareado e teste do sinal).

**RESULTADOS:**

Para o Sistema de Codificação da Atividade Facial Neonatal aplicado à beira do leito e em vídeo, os escores obtidos por A e B mostraram uma correlação intraclasse significante (0,62), sem presença de viés (teste t e do sinal: p > 0,05). Para a Escala de Conforto Clínico à beira do leito, os escores obtidos por A e B mostraram uma correlação significante (0,55), foi detectado: o escore obtido por A foi, em média, dois pontos superior ao de B (teste t e do sinal: p < 0,05). Para a mesma escala aplicada em vídeo, os escores obtidos por A e B não mostraram correlação (0,25) e detectou-se viés (teste t e do sinal: p < 0,05).

**CONCLUSÃO:**

Os resultados reforçam a confiabilidade do Sistema de Codificação da Atividade Facial Neonatal aplicado à beira do leito para a avaliação da dor no recém-nascido pré-termo.

## INTRODUCTION

When dealing with the assessment of pain, a differentiation must be made between evaluating and measuring the painful phenomenon. Measurement provides a value for the pain, whereas evaluation attempts to furnish the most complete picture possible of the phenomenon being studied, which may include its quantification, among other things.^1^ Among the important characteristics that an instrument created for assessing a subjective phenomenon must possess, the following can be highlighted: validity, sensitivity, format, internal consistency, reliability, and applicability.^1,2^

The terms reliability, consistency and reproducibility have been used in an interchangeable manner in the context of the psychometric characteristics of instruments for pain evaluation. However, the term reliability appears to be the most appropriate for defining to what extent a result obtained through the application of a given pain scale represents the true score.^1^ Faced with a pain evaluation by means of a given scale, it is possible that systematic or random errors may occur. Such errors may originate from factors linked to the patient, the environment, the observers and the evaluation instrument itself. The reliability of the scale will be determined by the individual variation in the expression of the items, evaluated in relation to this variation, plus the measuring errors.^1^ In other words, when the errors are close to zero, the reliability will approach 100%, and vice versa. The greater the reliability of the instrument studied is, the more it can be believed that the differences found between patients or groups of patients studied are due to individual variations or interventions made in one of the groups.

For the assessment of pain in newborns, unidimensional or multidimensional instruments are available. The former may consist of more than one indicator, but they evaluate a single type of response to pain or present a single approach towards the painful phenomenon, for example, a behavioral approach alone.^3^ Among these, the following should be cited: The Neonatal Facial Coding System;^4,5^ the Infant Body Coding System;^6^ the Clinical Scoring System;^7,8^ and the Modified Behavioral Pain Scale.^9^ On the other hand, multidimensional evaluation of pain is done by means of combinations of objective and subjective measurements. The use of multidimensional evaluation is particularly appropriate for individuals for whom self-reporting is compromised for some reason,^3^ which occurs among patients with cognitive and/or verbal deficiencies and children aged less than five years.^10^ Among the published multidimensional scales for the assessment of pain in the neonatal period, prominence is given to the Neonatal Infant Pain Scale;^11^ the Premature Infant Pain Profile;^13^ and the CRIES scale^12^: "CRIES" stands for crying, requires increased oxygen administration, increased vital signs, expression and sleeplessness.

The study of the psychometric characteristics of the unidimensional and multidimensional scales mentioned above still presents many gaps. Among other problems, there is incomplete information on various scales with regard to their content and criteria validity, inter-observer and intra-observer reliability and their applicability within clinical settings.^2,3^ In this context, the present investigation had the objective of contributing to the characterization of psychometric parameters for the unidimensional scales by evaluating the reliability of the Neonatal Facial Coding System and the Clinical Scoring System. Specifically, this study aimed to analyze the reproducibility of pain scores obtained by two different observers when applying the Neonatal Facial Coding System and the Clinical Scoring System to premature newborns.

## METHODS

After the approval of the research project by the Research Ethics Committee of Universidade Federal de São Paulo, this cross-sectional study included newborn infants according to the following inclusion criteria: written consent from the mother; gestational age less than 37 weeks; postnatal age between 12 and 48 hours of life; and newborns, with or without ventilatory support using a tracheal tube, who were clinically stable from a respiratory, hemodynamic and metabolic point of view at the time of the study and who had not received acute painful stimuli for at least 30 minutes prior to the experimental observation. An interval of 45 to 60 minutes was allowed to elapse between the last breast feeding and the start of the study, so that the patient was to be found calm and responsive.

The following newborns were excluded from the population analyzed: those whose mothers had made systemic use of opioids during the pregnancy and/or the birth labor and/or the delivery; newborns to whom muscle relaxants, analgesics and/or sedatives had been administered; patients with a prior diagnosis of grades III and IV intraventricular hemorrhage^14^ and malformations of the central nervous system; or those that were intubated or re-intubated during the four hours prior to the observation for the study.

After the inclusion in the study, the following neonatal data were registered: type of delivery and maternal anesthesia; birth weight in grams; gestational age in weeks; gender; Apgar score at one and five minutes; postnatal age, presence of mechanical ventilation through a tracheal tube and intravenous lines at the time of the study.

Two neonatologists made assessments of the following behavioral pain scales at the bedside for ten minutes: the Neonatal Facial Coding System^4^ (Table 1) and the Clinical Scoring System^7,8^ (Table 2). During this period, each of these doctors independently gave scores on each of the above scales at the first, fifth and tenth minutes of observation. The scales were always scored in the same order: first the Neonatal Facial Coding System, followed by the Clinical Scoring System. The neonatologists, one man and one woman with two years of residence in pediatrics and two years of specialization in neonatal intensive care did not talk to each other during the observation, nor were they aware of the scoring given by the other one. During these ten minutes, the neonates were filmed using a fixed video camera that was focused on the patient's face and body.

**Table 1:** Neonatal Facial Coding System^4,5^

	0 points	1 point
**Brow bulge**	Absent	Present
**Eye squeeze**	Absent	Present
**Deepened nasolabial furrow**	Absent	Present
**Open lips**	Absent	Present
**Stretched mouth**	Absent	Present
**Lip purse**	Absent	Present
**Taut tongue**	Absent	Present
**Chin quiver**	Absent	Present

**Table 2:** Clinical Scoring System^7,8^

	0 points	1 point	2 points
**Sleep**	Absent	Short naps	≥ 10 minutes
**Facial Expressiona**	Marked/Constant	< Marked/Intermittent	Calm & Relaxed
**Sucking**	Absent	Intermittent	Rhythmic
**Hyperreactivity**	Spontaneous	To stimulation	Absent
**Agitation**	Incessant	Moderate	Absent
**Hypertonicity**	Strong	Moderate	Absent
**Toes and fingers**	Flexed/Constant	< Flexed/Intermittent	Normal
**Consolability** a	Absent	Calm after 1 minute	Calm in < 1 min.

a *: double scoring. Maximum: 20 points.*

Three months after finishing the bedside observations, the same neonatologists analyzed the two behavioral pain scales using the videotapes. For each period of ten minutes of observation, the scales were applied at the first, fifth and tenth minute. For this, the frame-freeze and slow-motion facilities were utilized. Each neonatologist separately analyzed all the videos, without communication between the two of them regarding the scores obtained for the different patients. Again, the scales were always scored in the same order: first the Neonatal Facial Coding System, followed by the Clinical Scoring System.

The final score taken from the ten minutes of observation of each patient at the bedside and on the video films by each neonatologist was the median of the scores obtained for each item analyzed at the first, fifth and tenth minutes. Although patients did not receive any aversive or pleasant stimulation during observation periods, they would naturally have been able to change their behavioral states during the 10 minute study periods. Therefore, it was decided that the median values of the scores obtained at the different time points would be used to represent the average pain behavior of each patient during the observation period. This choice was made at the planning stage for the methods to be employed for answering the research question.

With regard to statistical analysis to verify the agreement between the two observers, the intraclass correlation coefficient and its statistical significance was utilized.^15^ The correlation^16^ was considered to be excellent when the value of the coefficient was greater than 0.75, good when the coefficient was in the range of 0.75 to 0.40, and poor when the correlation coefficient was less than 0.40. The paired t test and the signal test complemented this study. The paired t test^17^ was aimed at comparing the average scores given by the two observers, and the signal test^17^ was utilized to verify the existence of any systematic error, i.e. situations in which one observer always gave higher or lower scores than the other. Or in other words, the detection of the existence of any bias in the reproducibility of the observations was sought through these complementary tests.

## RESULTS

A total of 22 premature newborns were studied, with an average birth weight of 1,804 ± 584 grams, with a minimum of 720 and a maximum of 3,000 grams. Of the 22 patients, 6 (27%) had very low weight; 13 (59%) presented weights ranging from 1,500 to 2,500 grams and 3 (14%) were over 2,500 grams. The average gestational age was 34 ± 2 weeks, with a minimum of 29 and a maximum of 36 weeks. Six patients (27%) had gestational ages ≤ 32 weeks. Vaginal delivery occurred for 14 newborns (64%), with locoregional anesthesia being administered for the delivery, for 64% of the mothers of the patients studied. Apgar scores in the first and fifth minutes were 7 ± 1 and 9 ± 1, respectively. The postnatal age was 30 ± 12 hours of life, with a minimum of 12 and a maximum of 47 hours of life. Seven neonates (32%) were intubated and needed mechanical ventilation, and 14 (64%) had at least one vascular access.

With regard to the results obtained through observation of the Neonatal Facial Coding System by the two neonatologists at the bedside, it was seen that there was agreement between the observers in seven patients (32%). A disagreement between the two doctors by only one point occurred in seven newborns (32%), a disagreement by two points in three (14%), and by more than two points in five (22%) (Table 3). The statistical comparison of the Neonatal Facial Coding System scores by the two bedside observers demonstrated a good and significant intraclass correlation coefficient (coefficient 0.62; p = 0.0007), without differences between the average scoring by the two observers (difference – 0.77; p = 0.07) and an absence of bias between the observers (signal test: p = 0.19).

**Table 3:** Neonatal Facial Coding System scores at the bedside and in video films by the two observers (A and B) for the 22 patients (P1 – P22) studied

	P	P	P	P	P	P	P	P	P	P	P	P	P	P	P	P	P	P	P	P	P	P
	1	2	3	4	5	6	7	8	9	10	11	12	13	14	15	16	17	18	19	20	21	22
**BEDSIDE**																						
A	3	**3**	**5**	**3**	**6**	3	**1**	1	**2**	0	**1**	**4**	**0**	**2**	8	0	**1**	**0**	0	**1**	5	**1**
B	5	**2**	**5**	**4**	**7**	1	**1**	3	**2**	3	**2**	**5**	**0**	**3**	5	4	**0**	**0**	5	**1**	8	**1**
**VIDEO FILMS**																						
A	**1**	3	5	1	**5**	1	**1**	**1**	**1**	**0**	**0**	3	**0**	**2**	5	**0**	**0**	**0**	4	**1**	5	**1**
B	**2**	1	8	3	**4**	3	**2**	**2**	**0**	**1**	**1**	1	**0**	**2**	3	**1**	**0**	**0**	1	**0**	2	**2**

*Patients for whom there was concordance between the observers and disagreement of only one point are indicated in **bold***.

For the video assessment of the Neonatal Facial Coding System, agreement between the patients was verified in four neonates (18%). A disagreement between the doctors by one, two, or more points was demonstrated in 10 newborns (45%), five (23%) and three (14%), respectively (Table 3). The statistical comparison of the scoring obtained by the two observers for the Neonatal Facial Coding System from video film demonstrated a good and significant intraclass correlation coefficient (coefficient 0.61; p = 0.0008), without differences between the average scoring by the two observers (difference 0.05; p = 0.90) and an absence of bias between the observers (signal test: p = 0.81).

With regard to the results obtained through observation of the Clinical Scoring System by the two neonatologists at the bedside, it was seen that there was concordance between the observers in four patients (18%). A disagreement between the two doctors by only one point occurred in five newborns (23%), a disagreement by two points in six (27%), and by greater than two points in seven (32%) (Table 4). The statistical comparison of the scoring obtained for the Clinical Scoring System by the two bedside observers demonstrated a good and significant intraclass correlation coefficient (coefficient 0.55; p = 0.003), with an average difference of two points between the two observers, signifying that neonatologist ***A*** was, on average, systematically scoring two points higher than ***B*** (difference 2.05; p = 0.0002), with a significant bias between the observers (signal test: p = 0.001).

**Table 4:** Clinical Scoring System scores at the bedside and in video films by the two observers (A and B) for the 22 patients (P1 – P22) studied

	P	P	P	P	P	P	P	P	P	P	P	P	P	P	P	P	P	P	P	P	P	P
	1	2	3	4	5	6	7	8	9	10	11	12	13	14	15	16	17	18	19	20	21	22
**BEDSIDE**																						
A	**12**	13	**10**	**11**	10	15	15	11	10	12	**13**	11	**16**	13	11	12	**16**	**18**	**10**	14	**7**	14
B	**12**	11	**9**	**11**	6	10	9	9	8	8	**13**	4	**16**	10	8	10	**17**	**17**	**9**	12	**8**	12
**VIDEO FILMS**																						
A	15	12	10	12	10	15	16	14	11	14	15	**10**	**16**	13	11	14	**16**	**18**	14	17	7	**12**
B	10	9	3	16	8	10	9	8	17	10	9	**10**	**17**	10	8	12	**17**	**17**	8	12	9	**12**

*Patients for whom there was concordance between the observers and disagreement of only one point are indicated in **bold**.*

For the video assessment of the Clinical Scoring System, agreement was verified between the patients in two neonates (8%). A disagreement between the doctors by one, two, or more points was demonstrated in three newborns (14%), three (14%) and 14 (64%), respectively (Table 4). The statistical comparison of the scoring obtained for the Clinical Scoring System by the two observers from videos demonstrated an absence of correlation between the observers since the intraclass correlation coefficient was lower than 0.40 (coefficient 0.25; p = 0.12), with an average difference of two points between the two observers, signifying that neonatologist ***A*** was, on average, systematically scoring two points higher than ***B*** (difference 2.32; p = 0.007), with a significant bias between the observers (signal test: p = 0.04).

## DISCUSSION

When working with pain assessment instruments, the subjectivity of the person expressing the pain and the subjectivity of the professional assessing it need to be taken into account. This makes the elaboration of such instruments a fundamental matter for achieving a more homogenous therapeutic approach by the health team. In newborns, the existence of painful phenomena is inferred by adult observers by means of the physiological and behavioral responses to the nociceptive stimulus. Thus, in dealing with newborns, the elaboration and validation of such pain assessment instruments is crucial for making it possible to establish protocols and routines for analgesia of critically ill neonates. It was with this preoccupation that two behavioral pain scales were utilized in the present study, which sought to deepen the knowledge of one of their fundamental psychometric characteristics, the reliability of the methods.

In methodological terms, it is important to stress that the present investigation was not concerned with whether the newborn was really feeling pain. In this way, no additional potentially painful stimulus was inflicted on the patients, whereas their clinical condition varied. Thus, 20% of the neonates were on mechanical ventilation and had at least one intravenous line; another 20% needed at least one intravenous access; and around 60% consisted of premature newborns that were stable from a clinical point of view. In other words, the primary objective of the trial was to verify whether the behavioral pain scales applied to premature patients with or without pain presented consistent results when different individuals made the observations.

To this end, the intraclass correlation coefficient was employed as a support for the study. According to Bland and Altman^15^ and Johnston,^1^ the intraclass correlation coefficient is a more precise indicator of the reliability of the measure, as it is modeled to verify the variability of the subject in relation to the total variability of the scoring, by means of variance analysis. The intraclass correlation coefficient thus portrays the proportion of the score obtained that represents the true score, i.e. the reliability of the result. This portrayal is better than the reproducibility or concordance of results furnished by the methods routinely employed in scientific investigations that deal with pain scales. Furthermore, for a more correct interpretation of the data, the paired t test and the signal test complemented the intraclass correlation analysis. By utilizing these tests, detection of the presence of systematic errors and bias between the observers was sought.

It is known that the Neonatal Facial Coding System differentiates between newborns undergoing a painful procedure and those receiving a non-painful procedure. This occurs in full-term patients^5,18,20^ and in premature neonates,^19,20-25^ and when the Neonatal Facial Coding System evaluation is made from video film^5,20-24,26^ or at the bedside.^18,19,25^ These findings suggest that the scale has well-founded validity.^2^ Furthermore, a comparison of the Neonatal Facial Coding System with another scale for evaluation of facial movement has shown high correlation between the two, thereby also indicating its validity for the diagnosis of pain in neonates.^27^ With regard to the reliability of the Neonatal Facial Coding System, a study made by our group^18^ has demonstrated agreement between the observers for all items on the scale analyzed at the bedside. In the literature, this concordance has been described consistently, with kappa coefficients of 85% or more.^2^ The present investigation confirms and broadens these data. The results obtained with the Neonatal Facial Coding System have given evidence for good reliability of the scale, both at the bedside and on video film, as the intraclass correlation coefficients for both situations were close to 60% and there was no evidence for systematic error and/or bias between the observers. Moreover, this study contradicts another frequent affirmation in relation to the practical applicability of this scale: "unfortunately, the Neonatal Facial Coding System requires intensive training and hard work for its codification, rendering this scale of limited use in the daily routine of neonatal units".^2^ It can be perceived from this and other clinical trials^18,19,25,28^ that it is possible to simplify the application of the Neonatal Facial Coding System, observing the presence or absence of the facial movements described by Grunau and Craig^4^ and scoring their presence, with validity and reliability in the results obtained. Thus, this instrument may be useful for evaluating pain in newborns in clinical practice, and its employment may also be a tool for "teaching" health professionals to discern what the newborn wants to communicate by its facial movements.

On the other hand, the Clinical Scoring System^7,8^ is based on a combined view of a series of behaviors by newborns, including facial expressions, cries, motor activity, excitability, tonus, flexion of the extremities, and the quality of sucking. Its validity was suggested by Barrier et al.^8^ The former group verified the presence of higher scores among preverbal patients under analgesia in the postoperative period than among those that had not received analgesics, and the presence of an inverse relationship between their scores and the serum values of endorphins and catecholamines. Guinsburg et al.^28^ demonstrated that the Clinical Scoring System scores rose in premature intubated neonates that received a dose of fentanyl, but this did not occur in patients who received placebo. However, this fact was only demonstrated in the observation of video films, and it was not possible to achieve similar results at the bedside. Thus, despite results suggesting the validity of the scale for the evaluation of pain, no other psychometric property of the instrument had been studied.^2^ The present investigation has added new concerns about the clinical use of the Clinical Scoring System. Although the findings at the bedside gave evidence of good reliability for the scale (intraclass correlation coefficient of 55%), the complementary analyses, also at the bedside, demonstrated the presence of a systematic error between the observers and a bias. On the video films, the Clinical Scoring System was not shown to be a reliable instrument (the correlation coefficient was only 25%) and the presence of a systematic error between the observers and bias were also noted. In this way, this behavioral scale does not appear to be an instrument to be adopted for the evaluation of pain in neonatal units, as additional proof is needed for its validation and its reliability is poor.

## CONCLUSION

The measurement of a phenomenon forms part of the principles of scientific investigation, and it is impossible to manage any clinical problem without having a measure on which to base treatment.^29^ In this context, the results obtained here may help those who wish to utilize appropriate instruments for evaluating and treating pain in newborns. Prominence can be given to the application of the Neonatal Facial Coding System at the bedside, whose reliability was confirmed in the present study.
